# Application of environmental DNA analysis to inform invasive fish eradication operations

**DOI:** 10.1007/s00114-017-1453-9

**Published:** 2017-03-29

**Authors:** Phillip I. Davison, Gordon H. Copp, Véronique Créach, Lorenzo Vilizzi, J. R. Britton

**Affiliations:** 1 Centre for Environment, Fisheries and Aquaculture Science, Pakefield Road, Lowestoft, NR33 0HT UK; 2 Centre for Conservation and Environmental Change, School of Conservation Sciences, Bournemouth University, Poole, Dorset, BH12 5BB UK; 3 Environmental and Life Sciences Graduate Program, Trent University, Peterborough, ON K9J 7B8 Canada; 4 Department of Ecology and Vertebrate Zoology, Faculty of Biology and Environmental Protection, University of Łódź, 12/16 Banacha ul., 90-237 Łódź, Poland

**Keywords:** eDNA, Conventional PCR, Fish trapping, Non-native species management

## Abstract

Environmental DNA (eDNA) detection of non-native species has considerable potential to inform management decisions, including identifying the need for population control and/or eradication. An invasive species of European concern is the Asian cyprinid fish, topmouth gudgeon (*Pseudorasbora parva*)*.* Here, eDNA analyses were applied at a commercial angling venue in southern England to inform operations aiming to eradicate *P. parva*, which had only ever been observed in one of the venue’s seven unconnected angling ponds. Eradication of *P. parva* was initially attempted by repeated depletion of the population using fish traps (crayfish traps fitted with 5 mm mesh netting) and the introduction of native predators over a 4-year period. The very low number of *P. parva* captured following these eradication efforts suggested a possible population crash. Conventional PCR analysis of water samples using species-specific primers was applied to all seven ponds to confirm that *P. parva* was present in only one pond, that the eradication attempt had indeed failed and that the species’ distribution in the pond appeared to be restricted to three bankside locations. The continued presence of *P. parva* at these locations was confirmed by subsequent trapping. Water samples from an adjacent, unconnected stream were also analysed using the eDNA methodology, but no DNA of *P. parva* was detected. The results suggest that further management action to eradicate *P. parva* be focused on the pond shown to contain the isolated *P. parva* population and thereby eliminate the risk of further dispersal. This study is the first to apply eDNA analysis to assess the efficacy of an eradication attempt and to provide evidence that the species was unlikely to be present in the other ponds, thus reducing the resources needed to control the species.

## Introduction

Surveys based on the detection of environmental DNA (eDNA) are increasingly used to detect the presence of a broad range of taxonomic groups in aquatic environments, with particular applications to species of conservation concern and non-native species (Jerde et al. 2011; Rees et al. 2014; Thomsen and Willerslev 2015). This is because eDNA-based surveys, which collect DNA shed by an organism via urine, faeces, mucus and epidermal cells into the water, tend to have greater power to detect elusive and/or rare organisms than conventional sampling approaches, e.g. bluegill sunfish (*Lepomis macrochirus*) (Takahara et al. 2013). This increased effectiveness, combined with relatively low financial costs and reduced impact on the environment, demonstrates that eDNA methodologies have high potential for enhancing the management of invasive fish species (Rees et al. 2014; Bylemans et al. 2016). Applications so far have included distribution assessments (Takahara et al. 2013; Keskin 2014), monitoring surveys on invasion fronts (Jerde et al. 2013; Adrian-Kalchhauser and Burkhardt-Holm 2016) and the evaluation of population eradication attempts (Dunker et al. 2016).

Eradication of potentially harmful non-native species is considered a key component of invasive species management, particularly in rapid response scenarios (UK Defra 2008; Britton et al. 2011a; Genovesi et al. 2015). Attempts to eradicate non-native fish species often involve application of a piscicide, such as rotenone (Allen et al. 2006; Britton et al. 2008), even though this practice can have substantial impacts on non-target fauna (e.g. Finlayson et al. 2010; Billman et al. 2011). In some circumstances, such as isolated water bodies, it may be possible to eradicate a fish species through a drain-down and liming of the water body (Britton et al. 2008). Other options for controlling invasive fish populations include repeated cropping by netting, trapping or electric fishing, and biological control by stocking predators (Britton et al. 2008).

Topmouth gudgeon (*Pseudorasbora parva*), a native species in eastern Asia, is one of the most invasive freshwater fish species in Europe, having spread across most of the continent within decades of its accidental introduction to Romania in the 1960s as a contaminant of Asian carp consignments (Gozlan et al. 2010). It arrived in England by this introduction vector in the mid-1980s (Gozlan et al. 2002). Such is the threat posed by *P. parva*, in particular its role as a healthy host of the rosette agent *Spherotecum destruens* (Gozlan et al. 2005), that it is the target of a national eradication campaign, which aims to remove all 23 known UK populations by the end of 2017 (UK EA 2014; GBNNNS 2015). *P. parva* is one of just two fish species currently listed as being of European Union concern under Regulation (EU) no. 1143/2014, requiring EU member states to implement management and control measures (European Union 2014). Methods which have been successfully used to eradicate local topmouth gudgeon populations include rotenone treatments (Britton et al. 2008) and repeated removals (Copp et al. 2007). Also, there are instances elsewhere in Europe where *P. parva* have established a population in a water body, persisted for a short period (<10 years) and then disappeared entirely (Copp et al. 2007). This suggests that the species may be susceptible to recruitment failure and local extirpation where their population numbers are dramatically reduced by either natural or human-assisted means.

To facilitate this management programme, an attempt to eradicate a *P. parva* population from a pond on a commercial recreational angling venue in southern England was undertaken between 2011 and 2016 using depletion and biocontrol methods. Given the requirement of such eradication attempts to undergo thorough post-operation evaluations to measure their efficacy (Britton et al. 2011a), the aim of this study was to demonstrate the potential use of eDNA analysis as a complement to conventional sampling methodologies for assessing the efficacy of fish eradication attempts. Our specific objectives were to: (1) develop a statistically-robust eDNA sampling protocol for evaluating the *P. parva* eradication attempt; (2) assess the efficacy of the eradication attempt using conventional and eDNA methods; and (3) determine whether or not *P. parva* was likely, based on eDNA analysis results, to be present in any other water bodies at the site.

## Materials and methods

### Primer design and testing

Species-specific primers for *P. parva* were designed to amplify a 350-base-pair region of the mitochondrial gene encoding cytochrome *c* oxidase subunit 1 (COI): forward primer (5′-3) CCTCTTCCGGAGTAGAGGCT and reverse primer (5′-3) TAGGATTGGGTCTCCTCCCC (Davison et al. 2016). Primer specificity was tested in silico against sequences of all UK freshwater fishes, using NCBI Primer-BLAST (http://www.ncbi.nlm.nih.gov/tools/primer-blast/). The primers were also tested experimentally in conventional PCRs against DNA extracts (DNeasy Blood and Tissue Kit, Qiagen, Hilden, Germany) from fish species from the same family (Cyprinidae) that are likely to occur at the study site: common carp (*Cyprinus carpio*), common bream (*Abramis brama*), roach (*Rutilus rutilus*) and rudd (*Scardinius erythrophthalmus*). Conventional PCRs were conducted using 0.1 ng of genomic DNA and none of the triplicate PCRs showed amplification for any of these species.

Testing of primer efficiency and optimisation of the PCR protocol was undertaken using DNA extracted from dorsal muscle tissue samples of *P. parva*. These tests showed that the primers reliably amplified *P. parva* DNA at a quantity of 1.5 × 10^−2^ ng. The ability of the primers to detect *P. parva* DNA reliably from water samples was confirmed in aquarium trials (1 fish in 44-L tanks) and in a field survey conducted in ponds where the species was known to occur (Davison et al. 2016).

### Study site and field sampling protocol

**Fig. 1:**
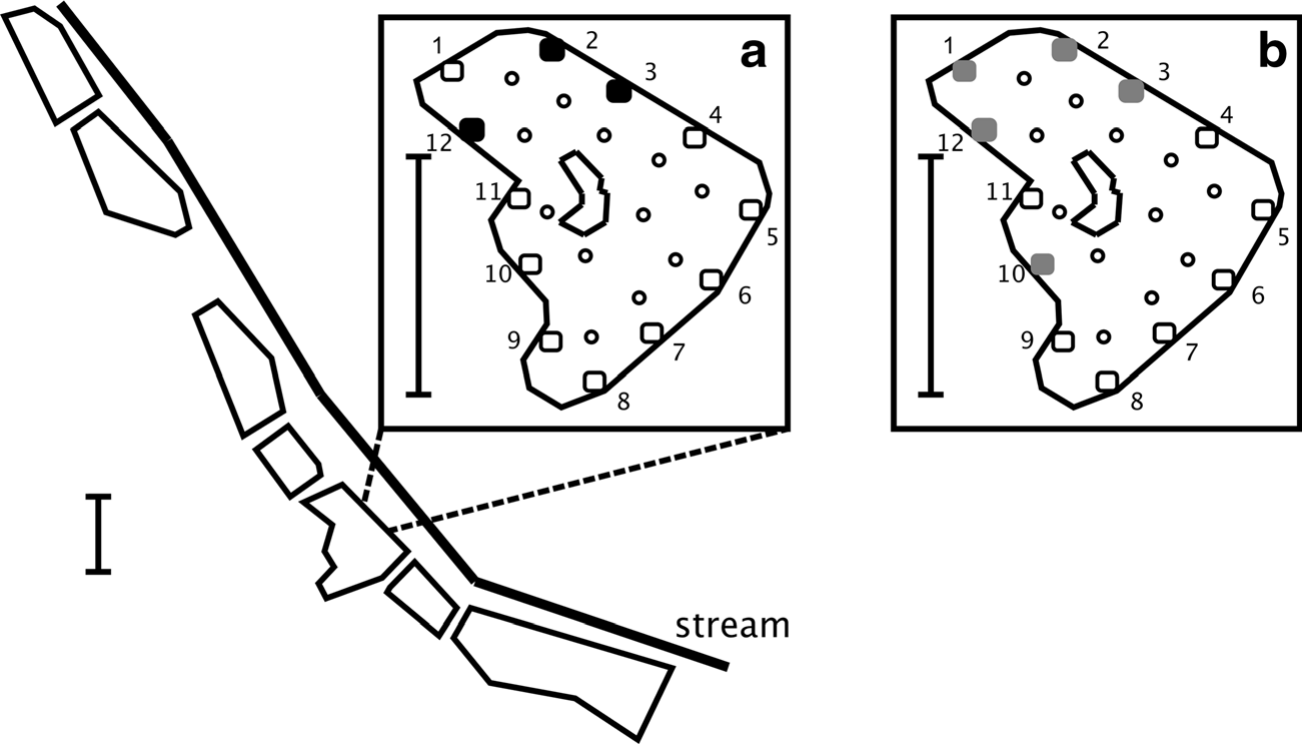
Schematic map (*scale bars* = 100 m) of the study site in the English county of Kent, showing location of the seven ponds and adjacent stream. In the infested lake (*inset maps*), pelagic sampling locations are indicated with *small open circles*, whereas littoral sampling locations (*squares*) are *numbered* (see Table 1), the *filled squares* indicating locations where positive detections of *P. parva* DNA occurred in the initial sampling survey (Sept 2014, *inset A*). Locations 1 and 10 also came up positive in Nov 2014 (*inset B*). See also Table 1

**Fig. 2:**
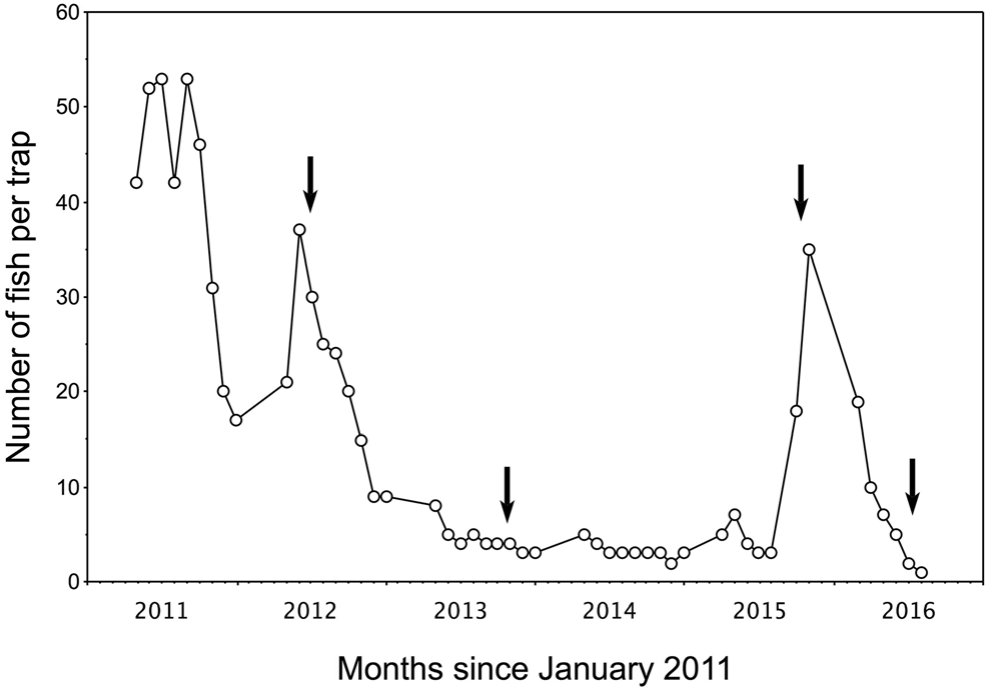
Numbers of topmouth gudgeon *Pseudorasbora parva*, calculated on a per trap per month basis, captured by fishery staff between 2011 and 2016 using fish traps (see ‘Materials and methods’) placed around the water body’s banks each sampling excursion. The *arrows* indicate dates of predator biocontrol release, i.e. 400, 200, 400 and 246 Eurasian perch *Perca fluviatilis* (*left to right*, respectively) of 6–9 cm total length

The recreational angling venue, which was located in Kent, South-east England (latitude 51° N, longitude 0° E), has no direct hydrological connections with an adjacent stream nor are any of the seven angling ponds connected (Fig. 1). A single specimen of *P. parva* was first captured in one of the angling ponds (area = 1.4 ha) in April 2004 but reported in the angling press to be a young grass carp *Ctenopharyngodon idella* (fishery owners, personal communication). An attempt to eradicate *P. parva* from this pond began in 2011 under the guidance of an independent fisheries consultant (commissioned by the fishery owners). From 2011 to July 2016, this consisted of intensive depletion using cylindrical fish traps (i.e. 60 by 30 cm crayfish traps with conical funnel entrance and fitted with 5 mm mesh netting). The depletion trapping was complemented by repeated, high density (116 kg ha^−1^) stocking of a native predatory fish, Eurasian perch (*Perca fluviatilis*)—a biocontrol method that has been demonstrated to exert a top-down effect on *P. parva* abundance (Davies and Britton 2015; Verhelst et al. 2016). Initial reports received by the authors indicated that by 2014, *P. parva* were no longer being captured; however, trapping data recently acquired from the venue’s owners revealed persistence of a very small number of *P. parva*, with the lowest capture densities occurring after predator releases (Fig. 2).

To ensure a statistically robust eDNA sampling protocol, an a priori power analysis was performed (http://homepage.stat.uiowa.edu/∼rlenth/Power/). This suggested that water should be collected from 24 sampling locations (12 littoral and 12 pelagic). At each sampling location, four sub-samples should be taken for analysis, and at least two PCR amplifications should be performed for each sub-sample. According to this protocol (corresponding to a doubly nested design), at a level of significance *α* = 0.05, statistical power (*β*) would equal 0.806 for the sampling zones, 0.978 for the water samples and 0.999 for the sub-samples.

Accordingly, post-eradication assessment using eDNA analysis consisted of three sampling steps (Sept 2014, Nov 2014, Feb–Mar 2015). Firstly, 24 1-L water samples were collected on 16 Sept 2014 in the infested pond, namely from 12 littoral zone locations spread equidistantly (40 m apart) around the pond shore and 12 from pelagic zone locations spaced around the water body (Fig. 1). Secondly, water sampling was undertaken during a return visit on 12 Nov 2014 at the six littoral sampling points in the infested water body closest to those where eDNA of *P. parva* was detected during step one. No *P. parva* DNA was detected in any of the 12 pelagic (mid-water) samples, so these pelagic sample locations were not considered further in the eDNA analysis. The water sampling on 12 Nov 2014 was complemented by intensive sampling, using the fish traps described above. Traps were deployed for 5 days in late Dec 2014, 10 days in early Feb and 6 days in early April 2015 (Fig. 2). Thirdly, water samples (1 L) were collected in 2015 on 17 Feb. 19 Feb and 5 March from 12 littoral zone locations in each of the other six ponds (areas of 0.5 to 2.4 ha), as well as at eight locations along the longitudinal course (1.5 km) of the small stream that runs adjacent to the ponds. Pelagic samples were not collected from the other six ponds, as this would have required movement of the boat between the water bodies, thus increasing the risk of cross contamination.

In all cases, water samples were collected using a 183-cm sampling pole with a 500-mL polypropylene sampling cup attached (Camlab Ltd., Cambridge, UK). The sampling cup was moved in a standardised manner from the bank (littoral samples) or boat (pelagic samples) to the greatest extent reached by the pole, ensuring no contact with the bottom sediment. At each sampling location, 1 L of water obtained using the sampling cup was poured into a sterilised plastic bottle. Samples were then placed in individual plastic bags and immediately refrigerated (4 °C) for transportation back to the laboratory. On each sampling day, two identical ‘blank samples’ (new sterilised bottles of de-ionised water from the laboratory), opened briefly in the field, were handled and transported in the same manner as the pond samples. Between samples, the sampling pole and cup were disinfected using Microsol 3+ (Anachem Ltd., Luton, UK) and washed with de-ionised water.

### Laboratory protocol

Within 24 h of collection, the water samples were filtered through a 0.4-μm pore size polycarbonate filter of diameter 47 mm (Isopore, EMD Millipore, Darmstadt, Germany) using a vacuum pump (EMD Millipore). From each sampling location, four sub-samples of 100 mL were filtered. Between filtration of samples from each location, the filtering equipment was sterilised using Microsol 3+ and washed with de-ionised water, and at regular intervals during filtration, de-ionised water was run through the filtration system, with these samples analysed to detect any potential cross contamination. The filters were immediately frozen at −80 °C. DNA extraction from the filters took place within 3 months from initial sampling using a PowerWater DNA Isolation Kit (MO BIO, Carlsbad, CA, USA).

Conventional PCR amplifications were performed in 20 μL reaction mixtures, containing 6 μL of DNA template, 0.5 μM of each primer, 10 μL (=50 units) HotStar Taq Plus DNA polymerase (Qiagen Fast Cycling PCR Kit) and 2 μL CoralLoad Fast Cycling Dye (Qiagen). The cycling conditions employed were an initial denaturation step at 95 °C for 5 min, followed by 32 cycles of denaturation (96 °C; 5 s), annealing (61 °C; 5 s) and extension (68 °C; 12 s), with a final extension at 72 °C for 1 min. Amplified PCR products were visualised using electrophoresis on 2% agarose gel, stained with SYBR Gold Nucleic Acid Gel Stain (Invitrogen, Paisley, UK). Three replicate PCRs were conducted for each 100 mL sub-sample, with each one including a negative control (de-ionised water) and a positive control (tissue-extracted *P. parva* DNA). To confirm the identity of sequences amplified from the pond samples, PCR products from the positive sampling points were purified (Nucleospin Gel and PCR Cleanup) and sequenced by a commercial service (Eurofins Genomic Services Ltd., Wolverhampton, UK).

To confirm that negative results were not detection errors (‘false negatives’) caused by PCR inhibition, additional PCRs were conducted using the PCR protocol described previously (Jane et al. 2015; Adrian-Kalchhauser and Burkhardt-Holm 2016). PCRs were performed using an eDNA sample (6 μL) from a single location within each pond that was spiked with 2 μL of genomic *P. parva* DNA (0.01 ng/μL). The strength of the resultant electrophoresis gel band was compared visually with that from the same quantity of *P. parva* DNA amplified in de-ionised water alone (i.e. without sample). As these PCRs indicated the presence of inhibition, a further set of PCRs were undertaken in which the extracted samples (one sub-sample from each sampling location) were re-analysed following a 1:5 dilution in de-ionised water, a technique used to combat inhibition by diluting the inhibitory compounds (McKee et al. 2015). Three replicate PCRs were conducted on these diluted samples. To assess whether inhibition was still occurring following the 1:5 dilution, three replicate PCRs per pond were conducted in which a spike of 0.02 ng of tissue-extracted *P. parva* DNA was added.

Filtration, extraction, PCR preparation and post-PCR analysis were undertaken in separate rooms of a laboratory dedicated to molecular biology, observing strict anti-contamination procedures (no transfer of equipment between rooms; changing of labcoats when moving between rooms; thorough cleaning of all equipment and surfaces before and after use).

## Results

In the initial sampling step, of the infested water body only, *P. parva* DNA was detected at 3 of the 12 littoral zone locations (Table 1). These sampling locations came from adjacent locations at one end of the pond (Fig. 1). DNA of *P. parva* was not detected in any of the 100-mL sample replicates collected from the pelagic zone. Spiking tests indicated a small level of inhibition occurring in pelagic and littoral samples. Two samples contained the minimum quantity of DNA required for sequencing, which confirmed the identity of the eDNA as that of *P. parva*. Both sequences showed a 100% match with 34 sequences of *P. parva* registered in the Genbank database (e.g. accession number HQ960448).

**Table 1:** Positive (+) and negative (−) detection of *P. parva* eDNA in water samples (initial sampling, 16 Sept 2014; repeat sampling, 12 Nov 2014) collected from the littoral zone of an angling pond in southern England

Sampling location	Initial sampling				Repeat sampling
	Sub-sample 1	Sub-sample 2	Sub-sample 3	Sub-sample 4	
1	−	−	−	−	+ (3)
2	−	−	+ (3)	+ (3)	+ (3)
3	−	−	−	+ (3)	+ (3)
4	−	−	−	−	n/a
5	−	−	−	−	n/a
6	−	−	−	−	n/a
7	−	−	−	−	n/a
8	−	−	−	−	n/a
9	−	−	−	−	n/a
10	−	−	−	−	+ (3)
11	−	−	−	−	−
12	−	−	+ (3)	+ (3)	+ (3)

Numbered sampling locations correspond to those in Fig. 1 (spacing = 40 m). Numbers in parentheses indicate number of conventional PCR replicates with positive detections, out of three performed on each sub-sample. Sub-sample number denotes the chronological order in which the four 100 mL sub-samples (from 1-L water samples) were analysed

*n/a* not applicable

In the second sampling step, repeat sampling and eDNA analysis of water from the locations where *P. parva* eDNA had been detected in step one provided further confirmation of the species’ presence. This corroborated the trapping data recently acquired from the venue’s owners (Fig. 2).

In the third sampling step, all sample replicates from the other six angling ponds and from the adjacent small stream proved negative for *P. parva* eDNA. Spiking tests indicated a small level of inhibition occurring in all six ponds. Following the 1:5 dilution of extracted samples to combat the detected inhibition, no further inhibition was detected. All samples that had previously shown negative for *P. parva* DNA (i.e. previously negative littoral locations and pelagic locations from the infested pond, and all samples from the a priori non-infested ponds) also proved to be negative following the 1:5 dilution. These results suggest that the level of inhibition occurring in the samples was not sufficient to mask the presence of DNA during the first analysis.

## Discussion

The current study demonstrates that eDNA surveys are a valuable method for post-evaluation of eradication attempts, with equal, if not greater, power to detect remnant populations of target species than conventional survey methods. Water samples subjected to eDNA analysis confirmed the persistence of a small population of *P. parva* in the infested pond, such as reported in detection studies elsewhere (Britton et al. 2011b). In the other water bodies, eDNA analysis corroborated trapping results for the other six angling ponds and electrofishing results for the adjacent stream, which indicate it is unlikely the species was present at the time of sampling.

Small-bodied fishes at low population densities can often be difficult to detect, and imperfect detection using conventional methods (i.e. electric fishing and trapping) has previously been demonstrated for *P. parva* in 100 m^2^ ponds (Britton et al. 2011b). At low population abundances, eDNA surveys may represent the most effective method of confirming the presence of a fish species. For example, eDNA sampling detected the presence of European weather loach (*Misgurnus fossilis*) in a location where it had not been recorded for 13 years using traditional methods, including fish traps, electrofishing and seine nets (Sigsgaard et al. 2015). In the present study, the spatial heterogeneity of the positive eDNA detections is likely to reflect the heterogeneous distribution of the target species, which has been recorded previously (Li et al. 2010; Davison et al. 2016). The lack of detections from the open water sampling locations is indicative of a distribution favouring shallow vegetated areas in the littoral zone (as previously shown for *P. parva*: Li et al. 2010) or an alternative favoured habitat type that is present in only a few isolated locations around the pond. The trapping of 78 specimens in the vicinity of these sampling points (7 months after the initial water sample collection), suggests that a small, localised population in this area was the most likely source of the detected eDNA.

Spatial heterogeneity of eDNA is common in lentic water bodies (e.g. Eichmiller et al. 2014), emphasising the need for sufficient water samples to be collected (with adequate spatial coverage) to increase the likelihood of detection of localised species in low abundance. In the present study, only five positive detections resulted from 96 sub-samples of water from 24 locations in the infested lake. Detection power could potentially have been improved by modifying the PCR protocol, such as increasing the number of cycles (Rameckers et al. 1997). The sensitivity of detection could arguably be increased by using quantitative real-time PCR (qPCR) protocols, for which higher levels of sensitivity have been reported (Tréguier et al. 2014; Biggs et al. 2015). However, in mesocosm trials, no difference between qPCR and conventional PCR was found in the detection of DNA of target species present at low density (Nathan et al. 2014). A practical consideration is that conventional PCR is financially less costly than qPCR, and therefore more likely to be available to those tasked with the management of invasive species (Davison et al. 2016).

The lack of detection of *P. parva* DNA in the six other lakes on site serves to corroborate the species’ absence in angler’s catches and conventional surveys undertaken before and after the eDNA survey (fishery owners, personal communication). Indeed, no *P. parva* were observed or captured in the adjacent stream during an electrofishing survey carried out a few months after the water samples for eDNA analysis were collected (Environment Agency, personal communication). Whilst caution is always needed when declaring a species to be absent on the basis of absence of detection, regardless of the survey method used (Mackenzie 2005; Kéry and Schmidt 2008), the statistically rigorous sampling protocol used here suggests that it is unlikely that *P. parva* is present in the other nearby, but unconnected, ponds and the stream. PCR-inhibiting compounds in the water are a potential cause of false negatives, but in this case study, the detected inhibition was not sufficient to affect the results. It does demonstrate, however, the importance of incorporating steps in laboratory protocols to assess the extent of inhibition, and if necessary to overcome inhibition by methods such as dilution of samples or addition of bovine serine albumin (Deiner et al. 2015; McKee et al. 2015).

The risk of false positives also needs to be considered when basing management decisions on the results of eDNA surveys. Positive detections should not necessarily be taken as an indication of presence of live organisms, as DNA could enter the water from other sources, e.g. decaying corpses or bird faeces (Merkes et al. 2014; Dunker et al. 2016). Before costly management action is taken, ‘ground truthing’ (i.e. capture of live individuals) is recommended to corroborate eDNA detection, such as was the case in the present study.

The present study demonstrates the applicability of eDNA surveys to assess the efficacy of eradication attempts in aquatic environments, providing additional support for studies elsewhere in which eDNA analysis was reported to be more sensitive than conventional methods for detecting species present in low abundance. Accurate assessments of the success of eradications is important; the continuation of a monitoring programme after the final individuals have been removed can be costly, whilst conversely the premature declaration of success and resultant cessation of monitoring can be even more costly and potentially nullify previous efforts (Rout et al. 2009, 2014). Surveys based on eDNA analysis are therefore an important tool to assist the decision-making process as regards the management of non-native species, both for early detection and rapid response, as well as for the assessment of eradication success. To this end, a nested quantitative PCR protocol is currently being tested in still and running waters for such applications to enhance the sensitivity of the analysis.
